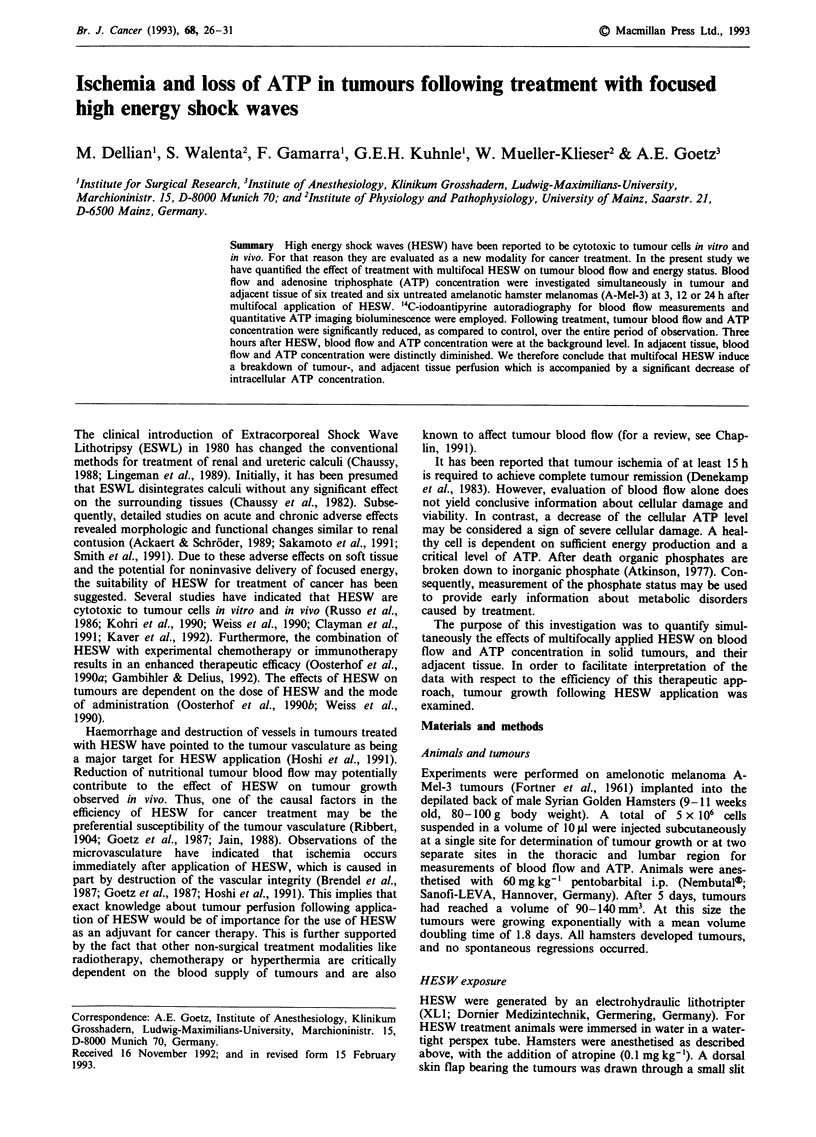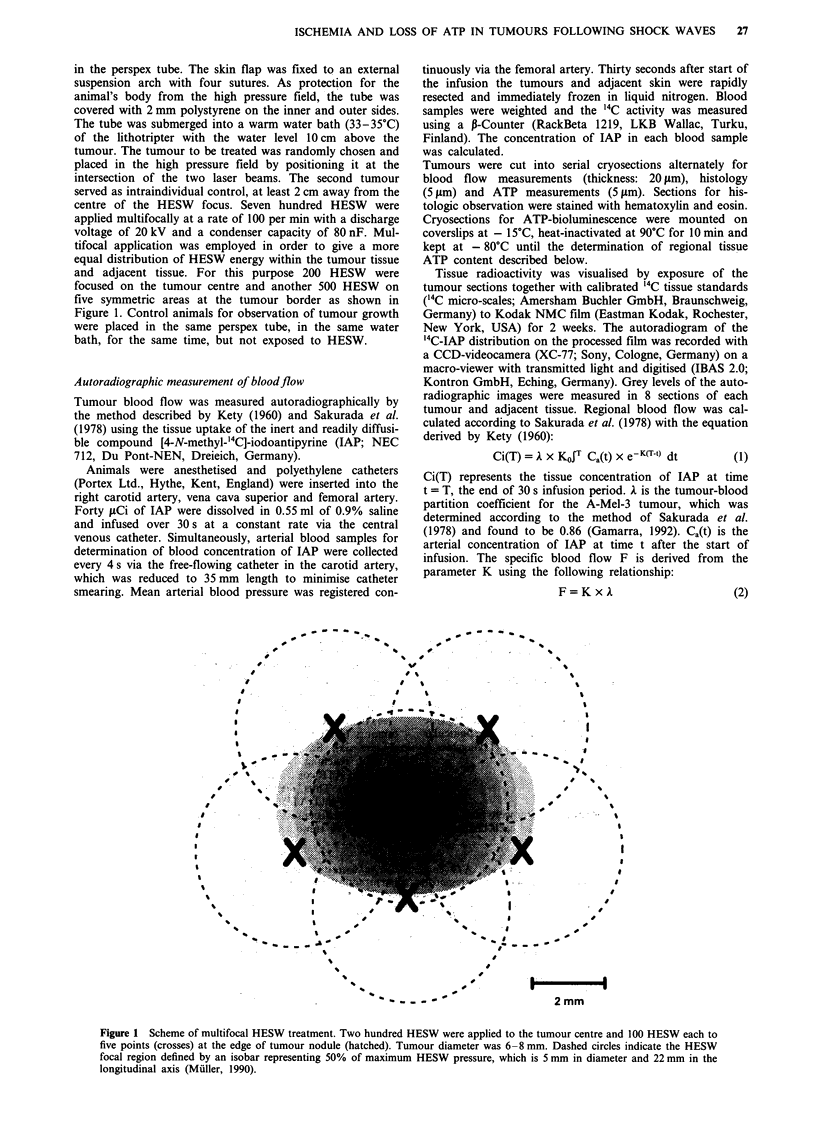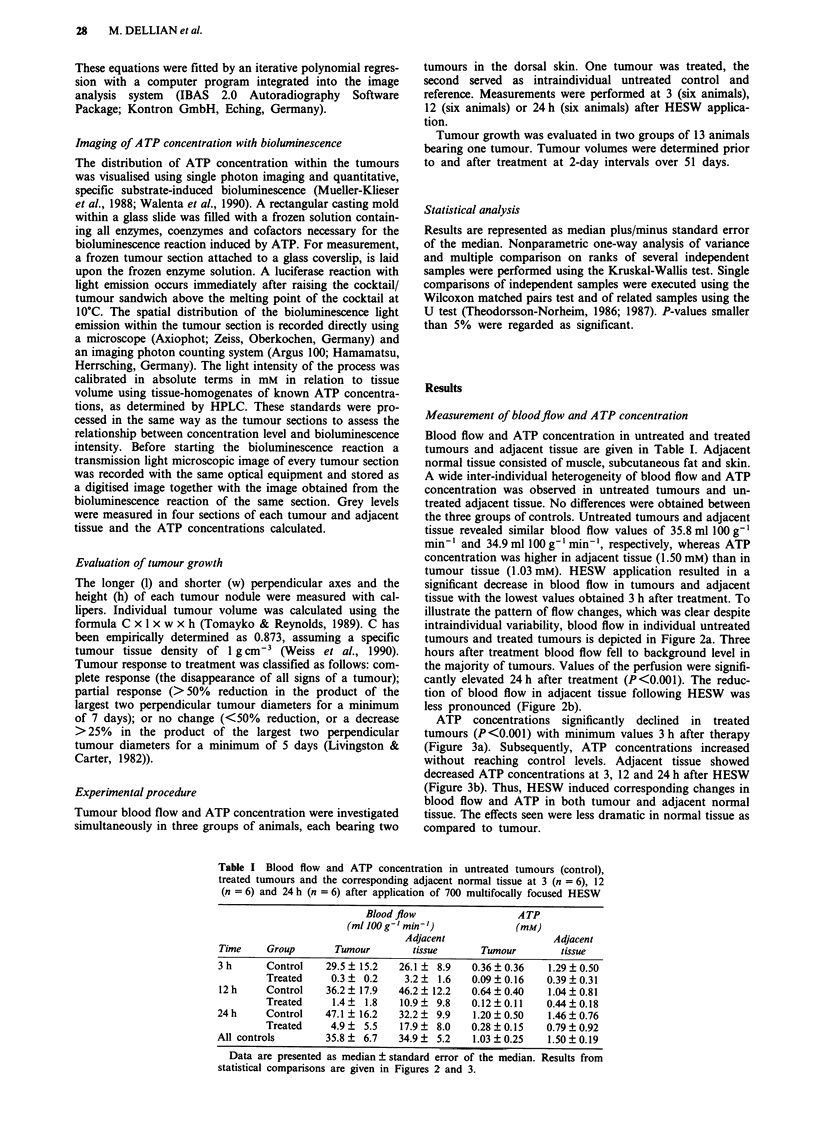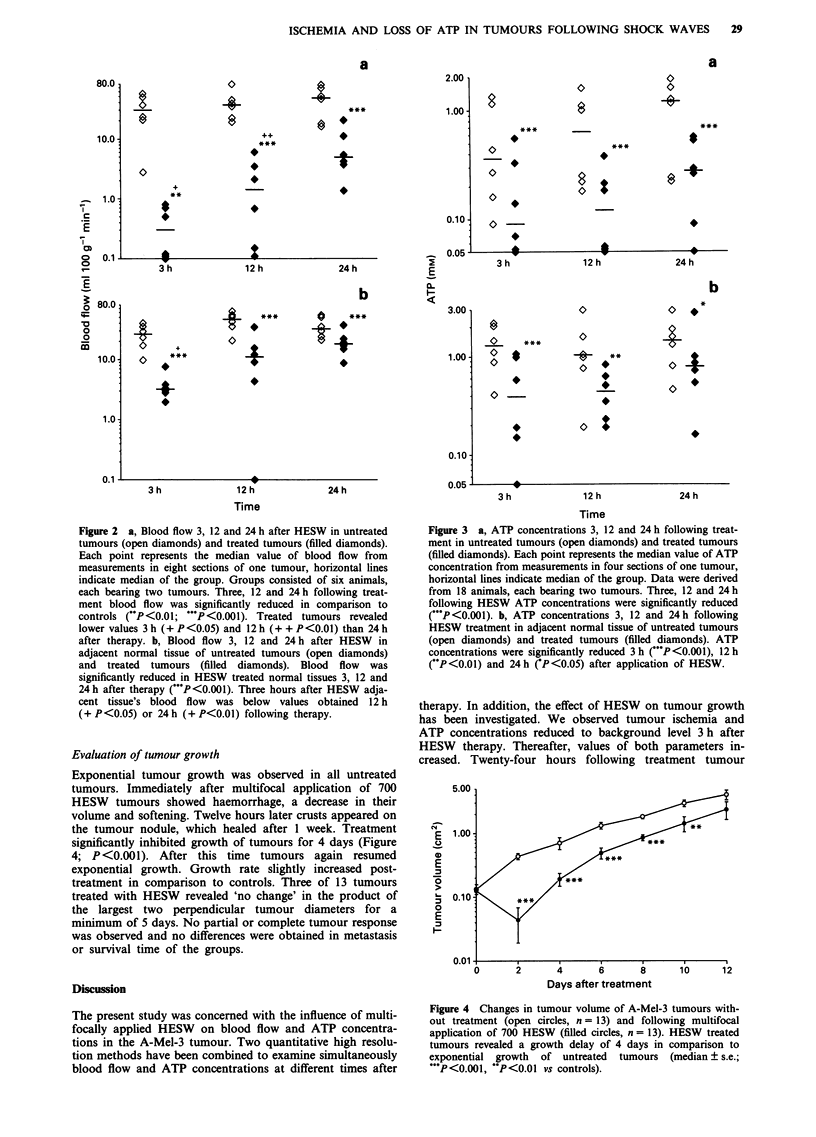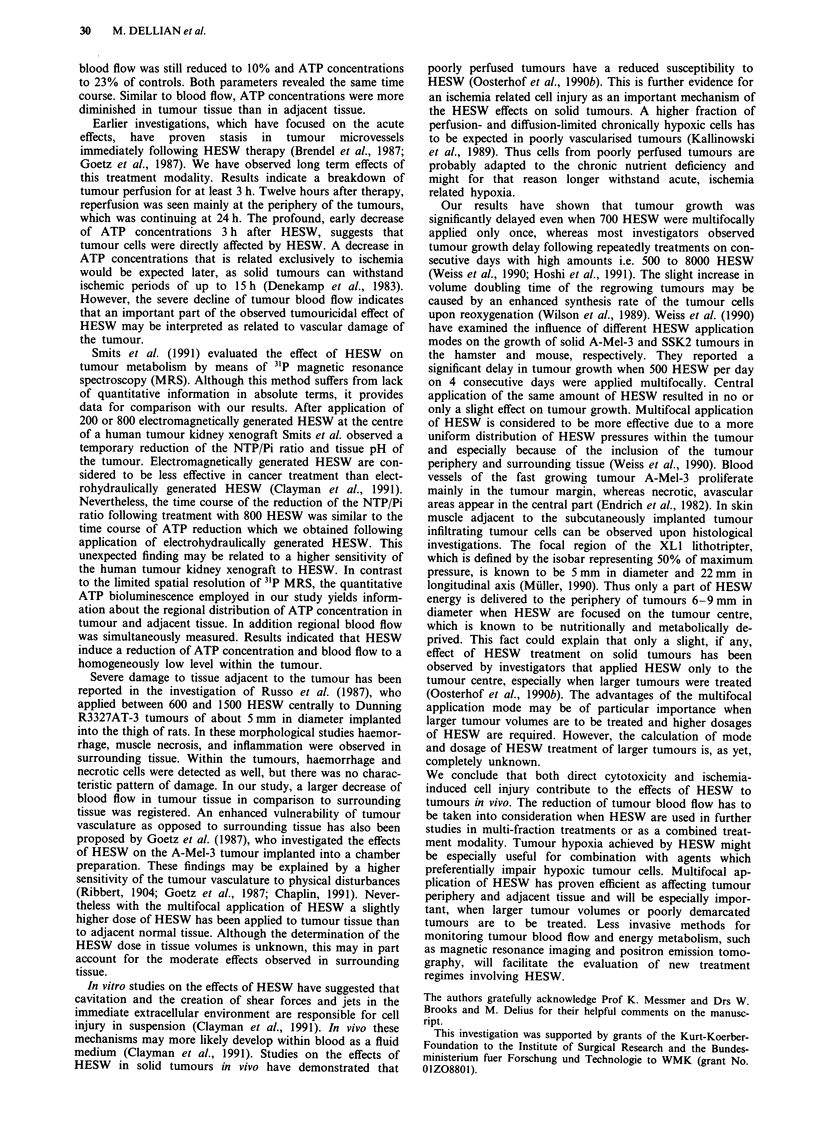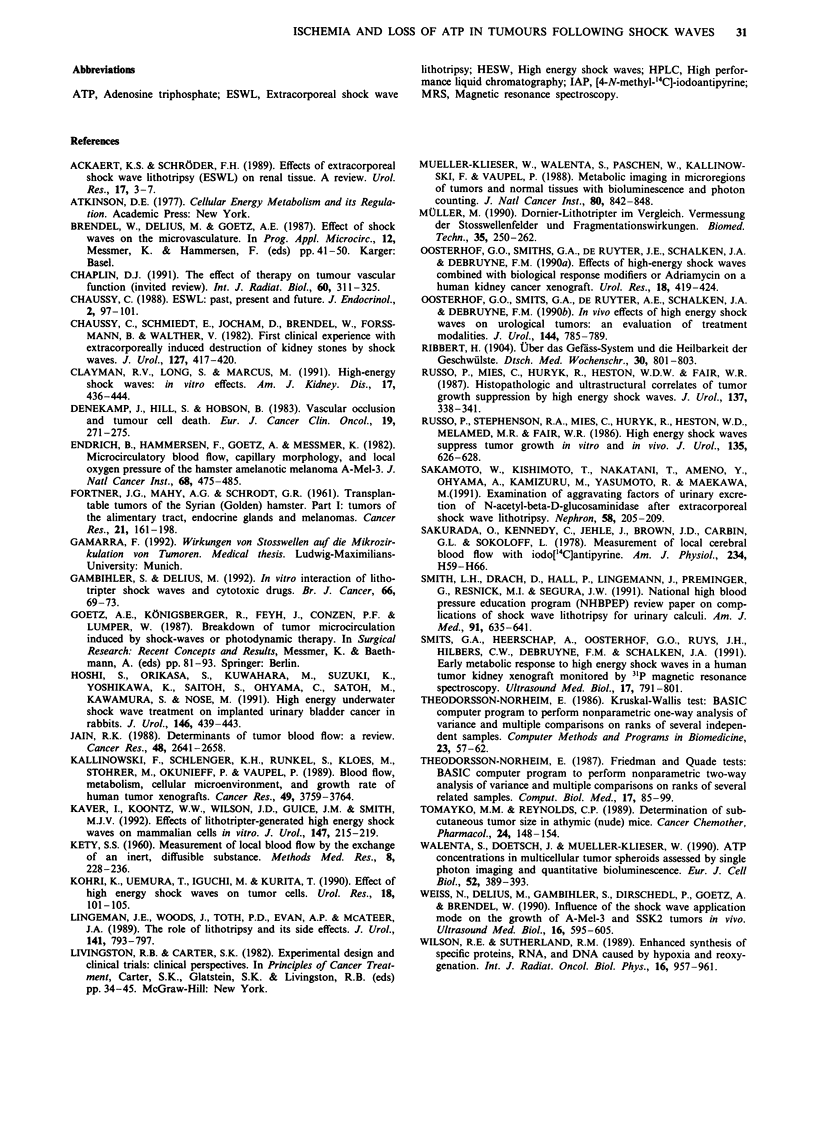# Ischemia and loss of ATP in tumours following treatment with focused high energy shock waves.

**DOI:** 10.1038/bjc.1993.281

**Published:** 1993-07

**Authors:** M. Dellian, S. Walenta, F. Gamarra, G. E. Kuhnle, W. Mueller-Klieser, A. E. Goetz

**Affiliations:** Institute for Surgical Research, Klinikum Grosshadern, Ludwig-Maximilians-University, Germany.

## Abstract

High energy shock waves (HESW) have been reported to be cytotoxic to tumour cells in vitro and in vivo. For that reason they are evaluated as a new modality for cancer treatment. In the present study we have quantified the effect of treatment with multifocal HESW on tumour blood flow and energy status. Blood flow and adenosine triphosphate (ATP) concentration were investigated simultaneously in tumour and adjacent tissue of six treated and six untreated amelanotic hamster melanomas (A-Mel-3) at 3, 12 or 24 h after multifocal application of HESW. 14C-iodoantipyrine autoradiography for blood flow measurements and quantitative ATP imaging bioluminescence were employed. Following treatment, tumour blood flow and ATP concentration were significantly reduced, as compared to control, over the entire period of observation. Three hours after HESW, blood flow and ATP concentration were at the background level. In adjacent tissue, blood flow and ATP concentration were distinctly diminished. We therefore conclude that multifocal HESW induce a breakdown of tumour-, and adjacent tissue perfusion which is accompanied by a significant decrease of intracellular ATP concentration.


Br. J. Cancer (1993), 68, 26-31 © Macmillan Press Ltd., 1993

### Ischemia and loss of ATP in tumours following treatment with focused high energy shock waves

M. Dellian[1], S. Walenta[2], F. Gamarra[1], G.E.H. Kuhnle[1], W. Mueller-Klieser[2] & A.E. Goetz[3]

*[1]Institute for Surgical Research, [3]Institute of Anesthesiology, Klinikum Grosshadern, Ludwig-Maximilians-University, Marchioninistr. 15, D-8000 Munich 70; and [2]Institute of Physiology and Pathophysiology, University of Mainz, Saarstr. 21, D-6500 Mainz, Germany.*

**Summary** High energy shock waves (HESW) have been reported to be cytotoxic to tumour cells *in vitro* and *in vivo*. For that reason they are evaluated as a new modality for cancer treatment. In the present study we have quantified the effect of treatment with multifocal HESW on tumour blood flow and energy status. Blood flow and adenosine triphosphate (ATP) concentration were investigated simultaneously in tumour and adjacent tissue of six treated and six untreated amelanotic hamster melanomas (A-Mel-3) at 3, 12 or 24 h after multifocal application of HESW. $^{14}$C-iodoantipyrine autoradiography for blood flow measurements and quantitative ATP imaging bioluminescence were employed. Following treatment, tumour blood flow and ATP concentration were significantly reduced, as compared to control, over the entire period of observation. Three hours after HESW, blood flow and ATP concentration were at the background level. In adjacent tissue, blood flow and ATP concentration were distinctly diminished. We therefore conclude that multifocal HESW induce a breakdown of tumour-, and adjacent tissue perfusion which is accompanied by a significant decrease of intracellular ATP concentration.

The clinical introduction of Extracorporeal Shock Wave Lithotripsy (ESWL) in 1980 has changed the conventional methods for treatment of renal and ureteric calculi (Chaussy, 1988; Lingeman *et al*., 1989). Initially, it has been presumed that ESWL disintegrates calculi without any significant effect on the surrounding tissues (Chaussy *et al*., 1982). Subsequently, detailed studies on acute and chronic adverse effects revealed morphologic and functional changes similar to renal contusion (Ackaert & Schröder, 1989; Sakamoto *et al*., 1991; Smith *et al*., 1991). Due to these adverse effects on soft tissue and the potential for noninvasive delivery of focused energy, the suitability of HESW for treatment of cancer has been suggested. Several studies have indicated that HESW are cytotoxic to tumour cells *in vitro* and *in vivo* (Russo *et al*., 1986; Kohri *et al*., 1990; Weiss *et al*., 1990; Clayman *et al*., 1991; Kaver *et al*., 1992). Furthermore, the combination of HESW with experimental chemotherapy or immunotherapy results in an enhanced therapeutic efficacy (Oosterhof *et al*., 1990*a*; Gambihler & Delius, 1992). The effects of HESW on tumours are dependent on the dose of HESW and the mode of administration (Oosterhof *et al*., 1990*b*; Weiss *et al*., 1990).

Haemorrhage and destruction of vessels in tumours treated with HESW have pointed to the tumour vasculature as being a major target for HESW application (Hoshi *et al*., 1991). Reduction of nutritional tumour blood flow may potentially contribute to the effect of HESW on tumour growth observed *in vivo*. Thus, one of the causal factors in the efficiency of HESW for cancer treatment may be the preferential susceptibility of the tumour vasculature (Ribbert, 1904; Goetz *et al*., 1987; Jain, 1988). Observations of the microvasculature have indicated that ischemia occurs immediately after application of HESW, which is caused in part by destruction of the vascular integrity (Brendel *et al*., 1987; Goetz *et al*., 1987; Hoshi *et al*., 1991). This implies that exact knowledge about tumour perfusion following application of HESW would be of importance for the use of HESW as an adjuvant for cancer therapy. This is further supported by the fact that other non-surgical treatment modalities like radiotherapy, chemotherapy or hyperthermia are critically dependent on the blood supply of tumours and are also known to affect tumour blood flow (for a review, see Chaplin, 1991).

It has been reported that tumour ischemia of at least 15 h is required to achieve complete tumour remission (Denekamp *et al*., 1983). However, evaluation of blood flow alone does not yield conclusive information about cellular damage and viability. In contrast, a decrease of the cellular ATP level may be considered a sign of severe cellular damage. A healthy cell is dependent on sufficient energy production and a critical level of ATP. After death organic phosphates are broken down to inorganic phosphate (Atkinson, 1977). Consequently, measurement of the phosphate status may be used to provide early information about metabolic disorders caused by treatment.

The purpose of this investigation was to quantify simultaneously the effects of multifocally applied HESW on blood flow and ATP concentration in solid tumours, and their adjacent tissue. In order to facilitate interpretation of the data with respect to the efficiency of this therapeutic approach, tumour growth following HESW application was examined.

### Materials and methods

### *Animals and tumours*

Experiments were performed on amelonotic melanoma A-Mel-3 tumours (Fortner *et al*., 1961) implanted into the depilated back of male Syrian Golden Hamsters (9-11 weeks old, 80-100 g body weight). A total of $5 \times 10^6$ cells suspended in a volume of 10 µl were injected subcutaneously at a single site for determination of tumour growth or at two separate sites in the thoracic and lumbar region for measurements of blood flow and ATP. Animals were anesthetised with 60 mg kg$^{-1}$ pentobarbital i.p. (Nembutal®; Sanofi-LEVA, Hannover, Germany). After 5 days, tumours had reached a volume of 90-140 mm$^3$. At this size the tumours were growing exponentially with a mean volume doubling time of 1.8 days. All hamsters developed tumours, and no spontaneous regressions occurred.

### *HESW exposure*

HESW were generated by an electrohydraulic lithotripter (XL1; Dornier Medizintechnik, Germering, Germany). For HESW treatment animals were immersed in water in a watertight perspex tube. Hamsters were anesthetised as described above, with the addition of atropine (0.1 mg kg$^{-1}$). A dorsal skin flap bearing the tumours was drawn through a small slit

Correspondence: A.E. Goetz, Institute of Anesthesiology, Klinikum Grosshadern, Ludwig-Maximilians-University, Marchioninistr. 15, D-8000 Munich 70, Germany.
Received 16 November 1992; and in revised form 15 February 1993.

in the perspex tube. The skin flap was fixed to an external suspension arch with four sutures. As protection for the animal's body from the high pressure field, the tube was covered with 2 mm polystyrene on the inner and outer sides. The tube was submerged into a warm water bath (33–35°C) of the lithotripter with the water level 10 cm above the tumour. The tumour to be treated was randomly chosen and placed in the high pressure field by positioning it at the intersection of the two laser beams. The second tumour served as intraindividual control, at least 2 cm away from the centre of the HESW focus. Seven hundred HESW were applied multifocally at a rate of 100 per min with a discharge voltage of 20 kV and a condenser capacity of 80 nF. Multifocal application was employed in order to give a more equal distribution of HESW energy within the tumour tissue and adjacent tissue. For this purpose 200 HESW were focused on the tumour centre and another 500 HESW on five symmetric areas at the tumour border as shown in Figure 1. Control animals for observation of tumour growth were placed in the same perspex tube, in the same water bath, for the same time, but not exposed to HESW.

### *Autoradiographic measurement of blood flow*

Tumour blood flow was measured autoradiographically by the method described by Kety (1960) and Sakurada *et al.* (1978) using the tissue uptake of the inert and readily diffusible compound [4-*N*-methyl-$^{14}$C]-iodoantipyrine (IAP; NEC 712, Du Pont-NEN, Dreieich, Germany).

Animals were anesthetised and polyethylene catheters (Portex Ltd., Hythe, Kent, England) were inserted into the right carotid artery, vena cava superior and femoral artery. Forty μCi of IAP were dissolved in 0.55 ml of 0.9% saline and infused over 30 s at a constant rate via the central venous catheter. Simultaneously, arterial blood samples for determination of blood concentration of IAP were collected every 4 s via the free-flowing catheter in the carotid artery, which was reduced to 35 mm length to minimise catheter smearing. Mean arterial blood pressure was registered continuously via the femoral artery. Thirty seconds after start of the infusion the tumours and adjacent skin were rapidly resected and immediately frozen in liquid nitrogen. Blood samples were weighted and the $^{14}$C activity was measured using a β-Counter (RackBeta 1219, LKB Wallac, Turku, Finland). The concentration of IAP in each blood sample was calculated.

Tumours were cut into serial cryosections alternately for blood flow measurements (thickness: 20 μm), histology (5 μm) and ATP measurements (5 μm). Sections for histologic observation were stained with hematoxylin and eosin. Cryosections for ATP-bioluminescence were mounted on coverslips at − 15°C, heat-inactivated at 90°C for 10 min and kept at − 80°C until the determination of regional tissue ATP content described below.

Tissue radioactivity was visualised by exposure of the tumour sections together with calibrated $^{14}$C tissue standards ($^{14}$C micro-scales; Amersham Buchler GmbH, Braunschweig, Germany) to Kodak NMC film (Eastman Kodak, Rochester, New York, USA) for 2 weeks. The autoradiogram of the $^{14}$C-IAP distribution on the processed film was recorded with a CCD-videocamera (XC-77; Sony, Cologne, Germany) on a macro-viewer with transmitted light and digitised (IBAS 2.0; Kontron GmbH, Eching, Germany). Grey levels of the autoradiographic images were measured in 8 sections of each tumour and adjacent tissue. Regional blood flow was calculated according to Sakurada *et al.* (1978) with the equation derived by Kety (1960):

$$Ci(T) = \lambda \times K_0\int^T C_a(t) \times e^{-K(T-t)}\,dt \qquad (1)$$

Ci(T) represents the tissue concentration of IAP at time t = T, the end of 30 s infusion period. λ is the tumour-blood partition coefficient for the A-Mel-3 tumour, which was determined according to the method of Sakurada *et al.* (1978) and found to be 0.86 (Gamarra, 1992). $C_a(t)$ is the arterial concentration of IAP at time t after the start of infusion. The specific blood flow F is derived from the parameter K using the following relationship:

$$F = K \times \lambda \qquad (2)$$

**Figure 1** Scheme of multifocal HESW treatment. Two hundred HESW were applied to the tumour centre and 100 HESW each to five points (crosses) at the edge of tumour nodule (hatched). Tumour diameter was 6–8 mm. Dashed circles indicate the HESW focal region defined by an isobar representing 50% of maximum HESW pressure, which is 5 mm in diameter and 22 mm in the longitudinal axis (Müller, 1990).

These equations were fitted by an iterative polynomial regression with a computer program integrated into the image analysis system (IBAS 2.0 Autoradiography Software Package; Kontron GmbH, Eching, Germany).

*Imaging of ATP concentration with bioluminescence*

The distribution of ATP concentration within the tumours was visualised using single photon imaging and quantitative, specific substrate-induced bioluminescence (Mueller-Klieser *et al.*, 1988; Walenta *et al.*, 1990). A rectangular casting mold within a glass slide was filled with a frozen solution containing all enzymes, coenzymes and cofactors necessary for the bioluminescence reaction induced by ATP. For measurement, a frozen tumour section attached to a glass coverslip, is laid upon the frozen enzyme solution. A luciferase reaction with light emission occurs immediately after raising the cocktail/tumour sandwich above the melting point of the cocktail at 10°C. The spatial distribution of the bioluminescence light emission within the tumour section is recorded directly using a microscope (Axiophot; Zeiss, Oberkochen, Germany) and an imaging photon counting system (Argus 100; Hamamatsu, Herrsching, Germany). The light intensity of the process was calibrated in absolute terms in mM in relation to tissue volume using tissue-homogenates of known ATP concentrations, as determined by HPLC. These standards were processed in the same way as the tumour sections to assess the relationship between concentration level and bioluminescence intensity. Before starting the bioluminescence reaction a transmission light microscopic image of every tumour section was recorded with the same optical equipment and stored as a digitised image together with the image obtained from the bioluminescence reaction of the same section. Grey levels were measured in four sections of each tumour and adjacent tissue and the ATP concentrations calculated.

*Evaluation of tumour growth*

The longer (l) and shorter (w) perpendicular axes and the height (h) of each tumour nodule were measured with callipers. Individual tumour volume was calculated using the formula $C \times l \times w \times h$ (Tomayko & Reynolds, 1989). C has been empirically determined as 0.873, assuming a specific tumour tissue density of $1\ g\ cm^{-3}$ (Weiss *et al.*, 1990). Tumour response to treatment was classified as follows: complete response (the disappearance of all signs of a tumour); partial response (>50% reduction in the product of the largest two perpendicular tumour diameters for a minimum of 7 days); or no change (<50% reduction, or a decrease >25% in the product of the largest two perpendicular tumour diameters for a minimum of 5 days (Livingston & Carter, 1982)).

*Experimental procedure*

Tumour blood flow and ATP concentration were investigated simultaneously in three groups of animals, each bearing two tumours in the dorsal skin. One tumour was treated, the second served as intraindividual untreated control and reference. Measurements were performed at 3 (six animals), 12 (six animals) or 24 h (six animals) after HESW application.

Tumour growth was evaluated in two groups of 13 animals bearing one tumour. Tumour volumes were determined prior to and after treatment at 2-day intervals over 51 days.

*Statistical analysis*

Results are represented as median plus/minus standard error of the median. Nonparametric one-way analysis of variance and multiple comparison on ranks of several independent samples were performed using the Kruskal-Wallis test. Single comparisons of independent samples were executed using the Wilcoxon matched pairs test and of related samples using the U test (Theodorsson-Norheim, 1986; 1987). P-values smaller than 5% were regarded as significant.

### Results

*Measurement of blood flow and ATP concentration*

Blood flow and ATP concentration in untreated and treated tumours and adjacent tissue are given in Table I. Adjacent normal tissue consisted of muscle, subcutaneous fat and skin. A wide inter-individual heterogeneity of blood flow and ATP concentration was observed in untreated tumours and untreated adjacent tissue. No differences were obtained between the three groups of controls. Untreated tumours and adjacent tissue revealed similar blood flow values of $35.8\ ml\ 100\ g^{-1}\ min^{-1}$ and $34.9\ ml\ 100\ g^{-1}\ min^{-1}$, respectively, whereas ATP concentration was higher in adjacent tissue (1.50 mM) than in tumour tissue (1.03 mM). HESW application resulted in a significant decrease in blood flow in tumours and adjacent tissue with the lowest values obtained 3 h after treatment. To illustrate the pattern of flow changes, which was clear despite intraindividual variability, blood flow in individual untreated tumours and treated tumours is depicted in Figure 2a. Three hours after treatment blood flow fell to background level in the majority of tumours. Values of the perfusion were significantly elevated 24 h after treatment ($P<0.001$). The reduction of blood flow in adjacent tissue following HESW was less pronounced (Figure 2b).

ATP concentrations significantly declined in treated tumours ($P<0.001$) with minimum values 3 h after therapy (Figure 3a). Subsequently, ATP concentrations increased without reaching control levels. Adjacent tissue showed decreased ATP concentrations at 3, 12 and 24 h after HESW (Figure 3b). Thus, HESW induced corresponding changes in blood flow and ATP in both tumour and adjacent normal tissue. The effects seen were less dramatic in normal tissue as compared to tumour.

**Table I** Blood flow and ATP concentration in untreated tumours (control), treated tumours and the corresponding adjacent normal tissue at 3 ($n = 6$), 12 ($n = 6$) and 24 h ($n = 6$) after application of 700 multifocally focused HESW

| | | *Blood flow ($ml\ 100\ g^{-1}\ min^{-1}$)* | | *ATP (mM)* | |
|---|---|---|---|---|---|
| *Time* | *Group* | *Tumour* | *Adjacent tissue* | *Tumour* | *Adjacent tissue* |
| 3 h | Control | 29.5 ± 15.2 | 26.1 ± 8.9 | 0.36 ± 0.36 | 1.29 ± 0.50 |
| | Treated | 0.3 ± 0.2 | 3.2 ± 1.6 | 0.09 ± 0.16 | 0.39 ± 0.31 |
| 12 h | Control | 36.2 ± 17.9 | 46.2 ± 12.2 | 0.64 ± 0.40 | 1.04 ± 0.81 |
| | Treated | 1.4 ± 1.8 | 10.9 ± 9.8 | 0.12 ± 0.11 | 0.44 ± 0.18 |
| 24 h | Control | 47.1 ± 16.2 | 32.2 ± 9.9 | 1.20 ± 0.50 | 1.46 ± 0.76 |
| | Treated | 4.9 ± 5.5 | 17.9 ± 8.0 | 0.28 ± 0.15 | 0.79 ± 0.92 |
| All controls | | 35.8 ± 6.7 | 34.9 ± 5.2 | 1.03 ± 0.25 | 1.50 ± 0.19 |

Data are presented as median ± standard error of the median. Results from statistical comparisons are given in Figures 2 and 3.

**Figure 2** a, Blood flow 3, 12 and 24 h after HESW in untreated tumours (open diamonds) and treated tumours (filled diamonds). Each point represents the median value of blood flow from measurements in eight sections of one tumour, horizontal lines indicate median of the group. Groups consisted of six animals, each bearing two tumours. Three, 12 and 24 h following treatment blood flow was significantly reduced in comparison to controls ($^{**}P<0.01$; $^{***}P<0.001$). Treated tumours revealed lower values 3 h (+ $P<0.05$) and 12 h (+ + $P<0.01$) than 24 h after therapy. b, Blood flow 3, 12 and 24 h after HESW in adjacent normal tissue of untreated tumours (open diamonds) and treated tumours (filled diamonds). Blood flow was significantly reduced in HESW treated normal tissues 3, 12 and 24 h after therapy ($^{***}P<0.001$). Three hours after HESW adjacent tissue's blood flow was below values obtained 12 h (+ $P<0.05$) or 24 h (+ $P<0.01$) following therapy.

*Evaluation of tumour growth*

Exponential tumour growth was observed in all untreated tumours. Immediately after multifocal application of 700 HESW tumours showed haemorrhage, a decrease in their volume and softening. Twelve hours later crusts appeared on the tumour nodule, which healed after 1 week. Treatment significantly inhibited growth of tumours for 4 days (Figure 4; $P<0.001$). After this time tumours again resumed exponential growth. Growth rate slightly increased post-treatment in comparison to controls. Three of 13 tumours treated with HESW revealed 'no change' in the product of the largest two perpendicular tumour diameters for a minimum of 5 days. No partial or complete tumour response was observed and no differences were obtained in metastasis or survival time of the groups.

### Discussion

The present study was concerned with the influence of multifocally applied HESW on blood flow and ATP concentrations in the A-Mel-3 tumour. Two quantitative high resolution methods have been combined to examine simultaneously blood flow and ATP concentrations at different times after

**Figure 3** a, ATP concentrations 3, 12 and 24 h following treatment in untreated tumours (open diamonds) and treated tumours (filled diamonds). Each point represents the median value of ATP concentration from measurements in four sections of one tumour, horizontal lines indicate median of the group. Data were derived from 18 animals, each bearing two tumours. Three, 12 and 24 h following HESW ATP concentrations were significantly reduced ($^{***}P<0.001$). b, ATP concentrations 3, 12 and 24 h following HESW treatment in adjacent normal tissue of untreated tumours (open diamonds) and treated tumours (filled diamonds). ATP concentrations were significantly reduced 3 h ($^{***}P<0.001$), 12 h ($^{**}P<0.01$) and 24 h ($^{*}P<0.05$) after application of HESW.

therapy. In addition, the effect of HESW on tumour growth has been investigated. We observed tumour ischemia and ATP concentrations reduced to background level 3 h after HESW therapy. Thereafter, values of both parameters increased. Twenty-four hours following treatment tumour

**Figure 4** Changes in tumour volume of A-Mel-3 tumours without treatment (open circles, $n = 13$) and following multifocal application of 700 HESW (filled circles, $n = 13$). HESW treated tumours revealed a growth delay of 4 days in comparison to exponential growth of untreated tumours (median ± s.e.; $^{***}P<0.001$, $^{**}P<0.01$ *vs* controls).

blood flow was still reduced to 10% and ATP concentrations to 23% of controls. Both parameters revealed the same time course. Similar to blood flow, ATP concentrations were more diminished in tumour tissue than in adjacent tissue.

Earlier investigations, which have focused on the acute effects, have proven stasis in tumour microvessels immediately following HESW therapy (Brendel *et al.*, 1987; Goetz *et al.*, 1987). We have observed long term effects of this treatment modality. Results indicate a breakdown of tumour perfusion for at least 3 h. Twelve hours after therapy, reperfusion was seen mainly at the periphery of the tumours, which was continuing at 24 h. The profound, early decrease of ATP concentrations 3 h after HESW, suggests that tumour cells were directly affected by HESW. A decrease in ATP concentrations that is related exclusively to ischemia would be expected later, as solid tumours can withstand ischemic periods of up to 15 h (Denekamp *et al.*, 1983). However, the severe decline of tumour blood flow indicates that an important part of the observed tumouricidal effect of HESW may be interpreted as related to vascular damage of the tumour.

Smits *et al.* (1991) evaluated the effect of HESW on tumour metabolism by means of $^{31}P$ magnetic resonance spectroscopy (MRS). Although this method suffers from lack of quantitative information in absolute terms, it provides data for comparison with our results. After application of 200 or 800 electromagnetically generated HESW at the centre of a human tumour kidney xenograft Smits *et al.* observed a temporary reduction of the NTP/Pi ratio and tissue pH of the tumour. Electromagnetically generated HESW are considered to be less effective in cancer treatment than electrohydraulically generated HESW (Clayman *et al.*, 1991). Nevertheless, the time course of the reduction of the NTP/Pi ratio following treatment with 800 HESW was similar to the time course of ATP reduction which we obtained following application of electrohydraulically generated HESW. This unexpected finding may be related to a higher sensitivity of the human tumour kidney xenograft to HESW. In contrast to the limited spatial resolution of $^{31}P$ MRS, the quantitative ATP bioluminescence employed in our study yields information about the regional distribution of ATP concentration in tumour and adjacent tissue. In addition regional blood flow was simultaneously measured. Results indicated that HESW induce a reduction of ATP concentration and blood flow to a homogeneously low level within the tumour.

Severe damage to tissue adjacent to the tumour has been reported in the investigation of Russo *et al.* (1987), who applied between 600 and 1500 HESW centrally to Dunning R3327AT-3 tumours of about 5 mm in diameter implanted into the thigh of rats. In these morphological studies haemorrhage, muscle necrosis, and inflammation were observed in surrounding tissue. Within the tumours, haemorrhage and necrotic cells were detected as well, but there was no characteristic pattern of damage. In our study, a larger decrease of blood flow in tumour tissue in comparison to surrounding tissue was registered. An enhanced vulnerability of tumour vasculature as opposed to surrounding tissue has also been proposed by Goetz *et al.* (1987), who investigated the effects of HESW on the A-Mel-3 tumour implanted into a chamber preparation. These findings may be explained by a higher sensitivity of the tumour vasculature to physical disturbances (Ribbert, 1904; Goetz *et al.*, 1987; Chaplin, 1991). Nevertheless with the multifocal application of HESW a slightly higher dose of HESW has been applied to tumour tissue than to adjacent normal tissue. Although the determination of the HESW dose in tissue volumes is unknown, this may in part account for the moderate effects observed in surrounding tissue.

*In vitro* studies on the effects of HESW have suggested that cavitation and the creation of shear forces and jets in the immediate extracellular environment are responsible for cell injury in suspension (Clayman *et al.*, 1991). *In vivo* these mechanisms may more likely develop within blood as a fluid medium (Clayman *et al.*, 1991). Studies on the effects of HESW in solid tumours *in vivo* have demonstrated that poorly perfused tumours have a reduced susceptibility to HESW (Oosterhof *et al.*, 1990*b*). This is further evidence for an ischemia related cell injury as an important mechanism of the HESW effects on solid tumours. A higher fraction of perfusion- and diffusion-limited chronically hypoxic cells has to be expected in poorly vascularised tumours (Kallinowski *et al.*, 1989). Thus cells from poorly perfused tumours are probably adapted to the chronic nutrient deficiency and might for that reason longer withstand acute, ischemia related hypoxia.

Our results have shown that tumour growth was significantly delayed even when 700 HESW were multifocally applied only once, whereas most investigators observed tumour growth delay following repeatedly treatments on consecutive days with high amounts i.e. 500 to 8000 HESW (Weiss *et al.*, 1990; Hoshi *et al.*, 1991). The slight increase in volume doubling time of the regrowing tumours may be caused by an enhanced synthesis rate of the tumour cells upon reoxygenation (Wilson *et al.*, 1989). Weiss *et al.* (1990) have examined the influence of different HESW application modes on the growth of solid A-Mel-3 and SSK2 tumours in the hamster and mouse, respectively. They reported a significant delay in tumour growth when 500 HESW per day on 4 consecutive days were applied multifocally. Central application of the same amount of HESW resulted in no or only a slight effect on tumour growth. Multifocal application of HESW is considered to be more effective due to a more uniform distribution of HESW pressures within the tumour and especially because of the inclusion of the tumour periphery and surrounding tissue (Weiss *et al.*, 1990). Blood vessels of the fast growing tumour A-Mel-3 proliferate mainly in the tumour margin, whereas necrotic, avascular areas appear in the central part (Endrich *et al.*, 1982). In skin muscle adjacent to the subcutaneously implanted tumour infiltrating tumour cells can be observed upon histological investigations. The focal region of the XL1 lithotripter, which is defined by the isobar representing 50% of maximum pressure, is known to be 5 mm in diameter and 22 mm in longitudinal axis (Müller, 1990). Thus only a part of HESW energy is delivered to the periphery of tumours 6–9 mm in diameter when HESW are focused on the tumour centre, which is known to be nutritionally and metabolically deprived. This fact could explain that only a slight, if any, effect of HESW treatment on solid tumours has been observed by investigators that applied HESW only to the tumour centre, especially when larger tumours were treated (Oosterhof *et al.*, 1990*b*). The advantages of the multifocal application mode may be of particular importance when larger tumour volumes are to be treated and higher dosages of HESW are required. However, the calculation of mode and dosage of HESW treatment of larger tumours is, as yet, completely unknown.

We conclude that both direct cytotoxicity and ischemia-induced cell injury contribute to the effects of HESW to tumours *in vivo*. The reduction of tumour blood flow has to be taken into consideration when HESW are used in further studies in multi-fraction treatments or as a combined treatment modality. Tumour hypoxia achieved by HESW might be especially useful for combination with agents which preferentially impair hypoxic tumour cells. Multifocal application of HESW has proven efficient as affecting tumour periphery and adjacent tissue and will be especially important, when larger tumour volumes or poorly demarcated tumours are to be treated. Less invasive methods for monitoring tumour blood flow and energy metabolism, such as magnetic resonance imaging and positron emission tomography, will facilitate the evaluation of new treatment regimes involving HESW.

The authors gratefully acknowledge Prof K. Messmer and Drs W. Brooks and M. Delius for their helpful comments on the manuscript.

This investigation was supported by grants of the Kurt-Koerber-Foundation to the Institute of Surgical Research and the Bundesministerium fuer Forschung und Technologie to WMK (grant No. 01ZO8801).

**Abbreviations**

ATP, Adenosine triphosphate; ESWL, Extracorporeal shock wave lithotripsy; HESW, High energy shock waves; HPLC, High performance liquid chromatography; IAP, [4-*N*-methyl-$^{14}$C]-iodoantipyrine; MRS, Magnetic resonance spectroscopy.

**References**

ACKAERT, K.S. & SCHRÖDER, F.H. (1989). Effects of extracorporeal shock wave lithotripsy (ESWL) on renal tissue. A review. *Urol. Res.*, **17**, 3–7.

ATKINSON, D.E. (1977). *Cellular Energy Metabolism and its Regulation.* Academic Press: New York.

BRENDEL, W., DELIUS, M. & GOETZ, A.E. (1987). Effect of shock waves on the microvasculature. In *Prog. Appl. Microcirc.*, **12**, Messmer, K. & Hammersen, F. (eds) pp. 41–50. Karger: Basel.

CHAPLIN, D.J. (1991). The effect of therapy on tumour vascular function (invited review). *Int. J. Radiat. Biol.*, **60**, 311–325.

CHAUSSY, C. (1988). ESWL: past, present and future. *J. Endocrinol.*, **2**, 97–101.

CHAUSSY, C., SCHMIEDT, E., JOCHAM, D., BRENDEL, W., FORSSMANN, B. & WALTHER, V. (1982). First clinical experience with extracorporeally induced destruction of kidney stones by shock waves. *J. Urol.*, **127**, 417–420.

CLAYMAN, R.V., LONG, S. & MARCUS, M. (1991). High-energy shock waves: *in vitro* effects. *Am. J. Kidney. Dis.*, **17**, 436–444.

DENEKAMP, J., HILL, S. & HOBSON, B. (1983). Vascular occlusion and tumour cell death. *Eur. J. Cancer Clin. Oncol.*, **19**, 271–275.

ENDRICH, B., HAMMERSEN, F., GOETZ, A. & MESSMER, K. (1982). Microcirculatory blood flow, capillary morphology, and local oxygen pressure of the hamster amelanotic melanoma A-Mel-3. *J. Natl Cancer Inst.*, **68**, 475–485.

FORTNER, J.G., MAHY, A.G. & SCHRODT, G.R. (1961). Transplantable tumors of the Syrian (Golden) hamster. Part I: tumors of the alimentary tract, endocrine glands and melanomas. *Cancer Res.*, **21**, 161–198.

GAMARRA, F. (1992). *Wirkungen von Stosswellen auf die Mikrozirkulation von Tumoren. Medical thesis.* Ludwig-Maximilians-University: Munich.

GAMBIHLER, S. & DELIUS, M. (1992). *In vitro* interaction of lithotripter shock waves and cytotoxic drugs. *Br. J. Cancer*, **66**, 69–73.

GOETZ, A.E., KÖNIGSBERGER, R., FEYH, J., CONZEN, P.F. & LUMPER, W. (1987). Breakdown of tumor microcirculation induced by shock-waves or photodynamic therapy. In *Surgical Research: Recent Concepts and Results*, Messmer, K. & Baethmann, A. (eds) pp. 81–93. Springer: Berlin.

HOSHI, S., ORIKASA, S., KUWAHARA, M., SUZUKI, K., YOSHIKAWA, K., SAITOH, S., OHYAMA, C., SATOH, M., KAWAMURA, S. & NOSE, M. (1991). High energy underwater shock wave treatment on implanted urinary bladder cancer in rabbits. *J. Urol.*, **146**, 439–443.

JAIN, R.K. (1988). Determinants of tumor blood flow: a review. *Cancer Res.*, **48**, 2641–2658.

KALLINOWSKI, F., SCHLENGER, K.H., RUNKEL, S., KLOES, M., STOHRER, M., OKUNIEFF, P. & VAUPEL, P. (1989). Blood flow, metabolism, cellular microenvironment, and growth rate of human tumor xenografts. *Cancer Res.*, **49**, 3759–3764.

KAVER, I., KOONTZ, W.W., WILSON, J.D., GUICE, J.M. & SMITH, M.J.V. (1992). Effects of lithotripter-generated high energy shock waves on mammalian cells *in vitro*. *J. Urol.*, **147**, 215–219.

KETY, S.S. (1960). Measurement of local blood flow by the exchange of an inert, diffusible substance. *Methods Med. Res.*, **8**, 228–236.

KOHRI, K., UEMURA, T., IGUCHI, M. & KURITA, T. (1990). Effect of high energy shock waves on tumor cells. *Urol. Res.*, **18**, 101–105.

LINGEMAN, J.E., WOODS, J., TOTH, P.D., EVAN, A.P. & MCATEER, J.A. (1989). The role of lithotripsy and its side effects. *J. Urol.*, **141**, 793–797.

LIVINGSTON, R.B. & CARTER, S.K. (1982). Experimental design and clinical trials: clinical perspectives. In *Principles of Cancer Treatment*, Carter, S.K., Glatstein, S.K. & Livingston, R.B. (eds) pp. 34–45. McGraw-Hill: New York.

MUELLER-KLIESER, W., WALENTA, S., PASCHEN, W., KALLINOWSKI, F. & VAUPEL, P. (1988). Metabolic imaging in microregions of tumors and normal tissues with bioluminescence and photon counting. *J. Natl Cancer Inst.*, **80**, 842–848.

MÜLLER, M. (1990). Dornier-Lithotripter im Vergleich. Vermessung der Stosswellenfelder und Fragmentationswirkungen. *Biomed. Techn.*, **35**, 250–262.

OOSTERHOF, G.O., SMITHS, G.A., DE RUYTER, J.E., SCHALKEN, J.A. & DEBRUYNE, F.M. (1990*a*). Effects of high-energy shock waves combined with biological response modifiers or Adriamycin on a human kidney cancer xenograft. *Urol. Res.*, **18**, 419–424.

OOSTERHOF, G.O., SMITS, G.A., DE RUYTER, A.E., SCHALKEN, J.A. & DEBRUYNE, F.M. (1990*b*). *In vivo* effects of high energy shock waves on urological tumors: an evaluation of treatment modalities. *J. Urol.*, **144**, 785–789.

RIBBERT, H. (1904). Über das Gefäss-System und die Heilbarkeit der Geschwülste. *Dtsch. Med. Wochenschr.*, **30**, 801–803.

RUSSO, P., MIES, C., HURYK, R., HESTON, W.D.W. & FAIR, W.R. (1987). Histopathologic and ultrastructural correlates of tumor growth suppression by high energy shock waves. *J. Urol.*, **137**, 338–341.

RUSSO, P., STEPHENSON, R.A., MIES, C., HURYK, R., HESTON, W.D., MELAMED, M.R. & FAIR, W.R. (1986). High energy shock waves suppress tumor growth *in vitro* and *in vivo*. *J. Urol.*, **135**, 626–628.

SAKAMOTO, W., KISHIMOTO, T., NAKATANI, T., AMENO, Y., OHYAMA, A., KAMIZURU, M., YASUMOTO, R. & MAEKAWA, M.(1991). Examination of aggravating factors of urinary excretion of N-acetyl-beta-D-glucosaminidase after extracorporeal shock wave lithotripsy. *Nephron*, **58**, 205–209.

SAKURADA, O., KENNEDY, C., JEHLE, J., BROWN, J.D., CARBIN, G.L. & SOKOLOFF, L. (1978). Measurement of local cerebral blood flow with iodo[$^{14}$C]antipyrine. *Am. J. Physiol.*, **234**, H59–H66.

SMITH, L.H., DRACH, D., HALL, P., LINGEMANN, J., PREMINGER, G., RESNICK, M.I. & SEGURA, J.W. (1991). National high blood pressure education program (NHBPEP) review paper on complications of shock wave lithotripsy for urinary calculi. *Am. J. Med.*, **91**, 635–641.

SMITS, G.A., HEERSCHAP, A., OOSTERHOF, G.O., RUYS, J.H., HILBERS, C.W., DEBRUYNE, F.M. & SCHALKEN, J.A. (1991). Early metabolic response to high energy shock waves in a human tumor kidney xenograft monitored by $^{31}$P magnetic resonance spectroscopy. *Ultrasound Med. Biol.*, **17**, 791–801.

THEODORSSON-NORHEIM, E. (1986). Kruskal-Wallis test: BASIC computer program to perform nonparametric one-way analysis of variance and multiple comparisons on ranks of several independent samples. *Computer Methods and Programs in Biomedicine*, **23**, 57–62.

THEODORSSON-NORHEIM, E. (1987). Friedman and Quade tests: BASIC computer program to perform nonparametric two-way analysis of variance and multiple comparisons on ranks of several related samples. *Comput. Biol. Med.*, **17**, 85–99.

TOMAYKO, M.M. & REYNOLDS, C.P. (1989). Determination of subcutaneous tumor size in athymic (nude) mice. *Cancer Chemother, Pharmacol.*, **24**, 148–154.

WALENTA, S., DOETSCH, J. & MUELLER-KLIESER, W. (1990). ATP concentrations in multicellular tumor spheroids assessed by single photon imaging and quantitative bioluminescence. *Eur. J. Cell Biol.*, **52**, 389–393.

WEISS, N., DELIUS, M., GAMBIHLER, S., DIRSCHEDL, P., GOETZ, A. & BRENDEL, W. (1990). Influence of the shock wave application mode on the growth of A-Mel-3 and SSK2 tumors *in vivo*. *Ultrasound Med. Biol.*, **16**, 595–605.

WILSON, R.E. & SUTHERLAND, R.M. (1989). Enhanced synthesis of specific proteins, RNA, and DNA caused by hypoxia and reoxygenation. *Int. J. Radiat. Oncol. Biol. Phys.*, **16**, 957–961.